# Energy and Nutrient Intake of Americans according to Meeting Current Dairy Recommendations

**DOI:** 10.3390/nu12103006

**Published:** 2020-09-30

**Authors:** Julie M. Hess, Christopher J. Cifelli, Victor L. Fulgoni III

**Affiliations:** 1 National Dairy Council, Rosemont, IL 60018, USA; Chris.Cifelli@dairy.org; 2 Nutrition Impact LLC, Battle Creek, MI 49014, USA; vic3rd@aol.com

**Keywords:** Dietary Guidelines for Americans, dietary guidance, dairy, dairy foods, nutrient adequacy

## Abstract

Most Americans do not meet dairy food recommendations from the 2015 Dietary Guidelines for Americans (DGA). This study assesses differences in nutrient intake between Americans who meet recommendations for dairy intake and those who do not, using data from the National Health and Nutrition Examination Survey from 2013–2014 and 2015–2016 (*n* = 5670 children ages 2–18 years and *n* = 10,112 adults ages 19+). Among children and adults, those meeting dairy food recommendations were significantly more likely to have adequate intake (% above Estimated Average Requirement (EAR)) of calcium, magnesium, phosphorus, riboflavin, vitamin A, vitamin B12, and zinc and consume above the Adequate Intake (AI) for potassium and choline than Americans not meeting dairy recommendations, regardless of age, sex, or race/ethnicity. Americans meeting dairy recommendations were also more likely to exceed recommendations for sodium and saturated fat but consume less added sugars. Nearly 60% of Americans 2 years and older not meeting dairy recommendations consumed calcium and magnesium below the EAR. Only about 20% of Americans who did not meet dairy recommendations consumed above the AI for potassium. Dairy foods make important and unique contributions to dietary patterns, and it can be difficult to meet nutrient needs without consuming recommended amounts of dairy foods.

## 1. Introduction

The 2015–2020 Dietary Guidelines for Americans (DGA) recommends that American children and adults consume dairy foods daily: 2 cup-equivalent servings of low-fat or fat-free milk, cheese, yogurt, or fortified soy beverage for children 2–3 years, 2.5 servings for children 4–8 years, and 3 servings for adolescents 9 to 18 years and all adults as part of the Healthy U.S.—Style and Healthy Vegetarian Eating Patterns [1]. The DGA bases these recommendations on the nutrient density of foods in the dairy group and their contributions to healthy eating patterns that remain within calorie, saturated fat, and sodium limits. The 2015 DGA identified the dairy group for its key contributions of calcium, phosphorus, vitamin A, vitamin D, riboflavin, vitamin B12, protein, potassium, zinc, choline, magnesium, and selenium in the diet [1]. According to food pattern modeling studies, the amounts of certain nutrients—calcium, magnesium, iron, vitamin A, riboflavin, potassium, vitamin D, and choline—in healthy eating patterns drop when dairy foods are removed from the diet. Calcium intake decreases by 68 to 88 percent, and vitamin D intake decreases by 20 to 30 percent across all age and sex groups without dairy in the diet [2].

Most Americans do not meet recommendations for dairy intake [1]. Data from the National Health and Nutrition Examination Survey (NHANES) from 2015–2016 indicate that 88 percent of the U.S. population consumes too few servings of dairy foods [3]. The only age group generally meeting dairy group recommendations is children 2–3 years, and consumption tends to fall with age [3]. Adults ages 20 years and older consume, on average, just 1.5 cup-equivalents of dairy foods per day [3]. While there are many possible reasons that Americans may not meet recommendations for dairy foods, including education, food security, and perceptions of dairy’s impact on health, milk allergy, and lactose intolerance may also be reasons for underconsuming dairy foods. Most common among young children, milk allergy causes an immune response to milk proteins that can lead to several different symptoms and requires avoiding dairy foods [4,5]. In the case of milk allergy, fortified soy beverage is an appropriate choice from the DGA’s “dairy group” to meet recommendations. Lactose intolerance occurs when individuals do not produce enough lactase and can cause discomfort such as bloating and diarrhea after consuming some lactose-containing dairy foods [6]. Based on self-report measures in a national sample of European American, African American, and Hispanic American adults, lactose intolerance affects approximately about 12% of Americans [7]. Lactose intolerance can be managed for most people by selecting lactose-free or reduced-lactose dairy foods [8].

Consuming less than the recommended servings of dairy foods can lead to inadequate intake of certain nutrients. The 2015 DGA lists the following ten nutrients as “shortfall nutrients” for the U.S. population: vitamin A, vitamin D, vitamin E, vitamin C, calcium, iron (for certain age/sex groups), magnesium, choline, potassium, and fiber. It further designates calcium, potassium, vitamin D, and fiber as “nutrients of public health concern” for the U.S. population, because their low intake “may pose a substantial public health concern” [1]. Other nutrients, including saturated fats, added sugars, and sodium are also described as nutrients of concern for Americans due to consumption rates higher than recommendations [1]. Dairy foods are an important source of several shortfall nutrients, including 3 of the 4 nutrients of public health concern, calcium, potassium, and vitamin D, indicating the importance of these foods to the nutrient density of the American diet. Milk was the top food source of calcium, potassium, and vitamin D among children 2–18 years and adults 19 and older in NHANES 2011–2014 [9] and NHANES 2003–2006 [10], respectively. Depending on products selected, however, dairy foods can also be sources of saturated fats, added sugars, and sodium.

Previous modeling work has indicated that increasing dairy food consumption to meet DGA recommendations could improve population-wide adequacy of some vitamins and minerals without increasing intake of saturated fats, added sugars, or sodium [11]. The aim of this study was to evaluate current dairy food group intakes of the U.S. population and the nutrient adequacy of those Americans who met recommendations for dairy foods compared to those Americans who did not meet dairy recommendations by using NHANES data from 2013–2016. These data were also evaluated by age, sex, and ethnicity. Our hypothesis is that Americans who meet recommendations for dairy food intake are more likely to meet nutrient needs than those who do not meet recommendations.

## 2. Materials and Methods

### 2.1. Data Source and Study Population

This analysis used data from day 1 of NHANES 2013–2014 and 2015–2016 (*n* = 15,782 after exclusions, including *n* = 5670 children 2–18 years and *n* = 10,112 adults 19+ years), which is a nationally representative sample of all noninstitutionalized Americans with over sampling of certain demographic groups. Data from pregnant and lactating females as well as participants with incomplete data, as determined by USDA, were excluded from analyses. Separate analyses were conducted for children 2–18 years, adults 19+ years, and all Americans 2+ years of age. Analyses were conducted for the overall population, for males/females separately, and for race/ethnic groups that were sampled in NHANES to provide nationally representative results: all, Hispanic, non-Hispanic Black, non-Hispanic White, and non-Hispanic Asian. Recommended servings of dairy foods were taken from the 2015–2020 DGA (Table 1).

### 2.2. Dietary Intake Assessment/Data

Intakes of energy and nutrients were from the NHANES total nutrient intake files which used the Food and Nutrient Database for Dietary Studies (FNDDS) for 2013–2014 and 2015–2016 to assess intakes [12,13]. The respective Food Patterns Equivalents Database (FPED) was used for assessing total dairy intake [14,15].

Usual intakes for total dairy and nutrients were determined using the National Cancer Institute (NCI) method [16]. The NCI macros (Mixtran and Distrib) were used to generate parameter effects after covariate adjustments (day of the week of the 24 h recall, coded as weekend (Friday–Sunday) or weekday (Monday–Thursday) and sequence of dietary recalls (first or second)) and to estimate the distribution of usual intake. Variance estimates were obtained using the two days of intake with one-day sampling weights. The one-part NCI model was used for total dairy and nutrients since these substances are consumed on most days by most subjects.

Population groups meeting dairy recommendations and population groups not meeting dairy recommendations were established with the first day of dietary recall (in-person interview). Usual intakes and percent population below the Estimated Average Requirement (EAR) or above Adequate Intakes (AI) were calculated and compared between population groups meeting dairy recommendations and population groups not meeting dairy recommendations. The nutrients assessed in this study included the 12 nutrients that the 2015 DGA identifies as key contributions from the dairy group (calcium, phosphorus, vitamin A, vitamin D, riboflavin, vitamin B12, protein, potassium, zinc, choline, magnesium, and selenium) as well as added sugars, sodium, and saturated fat [1]. For added sugars and saturated fat, the recommended intake was set to 10% of calories of saturated fat and 10% of calories of added sugars, in line with recommendations in the 2015 DGA [1].

### 2.3. Other Variables

Other demographic factors assessed included poverty income ratio (PIR; PIR < 1.35; 1.35≤ PIR ≤ 1.85; PIR > 1.85), physical activity level (sedentary, moderate, vigorous), overweight or obesity, energy intake (kcal), grams of food consumed, and BMI or BMI z-score. Physical activity was divided into 3 groups based on the number of days in which vigorous exercise was performed, using the Physical Activity Questionnaire responses: sedentary, 0–3 days per week; moderate, 4–6 days per week; and vigorous, 7 days per week.

### 2.4. Statistical Analyses

All analyses were adjusted for the complex sample design of NHANES using appropriate survey weights, strata, and primary sampling units. All statistical analyses were performed with SAS software (version 9.4; SAS Institute Inc., Cary, NC, USA). Differences in demographics of population groups meeting dairy recommendations and population groups not meeting dairy recommendations were assessed using *t*-tests. Differences in the percentage of the population meeting dietary recommendations for those meeting dairy recommendations and those not meeting dairy recommendations were assessed using z-scores. Significance was set at *p* < 0.05.

## 3. Results

Most children and adults did not meet dairy food intake recommendations in NHANES 2013–2016. Approximately one-fourth (27.8 ± 0.11%) of children ages 2–18 and just 13.6 ± 0.5% of adults age 19 and older consumed the recommended number of daily dairy servings.

Children 2–18 years who met dairy food recommendations tended to be younger, male, and non-Hispanic White with ”vigorous” activity levels and were less likely to be below a poverty-income ratio (PIR) of 1.35, have ”moderate” activity level, be non-Hispanic black, or overweight or obese (Table 2). These children also consumed more energy (kcal) and food (g) but had lower body mass index (BMI) z-scores (Table 3).

Similarly, adults (19+ years) who met dairy food recommendations were more likely to be younger, male, non-Hispanic white, with ”vigorous” activity levels and were less likely to have a ”moderate” activity level, be non-Hispanic black, overweight or obese. Adults meeting recommendations were also less likely to be non-Hispanic Asian and consumed more energy and food but with a lower BMI. There were no differences in PIR between adults meeting versus not meeting dairy food recommendations (Table 2).

Adults and children (2+ years, females and males) meeting dairy food recommendations were less likely to be below the EAR for calcium, magnesium, phosphorus, protein, riboflavin, vitamin A, vitamin B12, vitamin D, selenium, and zinc than those not meeting dairy food recommendations (Table 2). A larger percentage of the population meeting dairy recommendations was also above the AI for potassium (71.5%) (*p* < 0.0001) and choline (36.9%) (*p* < 0.0001) compared to those not meeting dairy recommendations. A larger percentage of the population not meeting dairy recommendations consumed more than 10% of calories from added sugars (64%) (*p* = 0.0021). Americans meeting dairy recommendations were, however, more likely to exceed 10% of calories from saturated fat (*p* < 0.0001) and to consume above the AI for sodium (*p* < 0.0001) than those who did not meet dairy recommendations. Population inadequacy (% below EAR) for calcium, phosphorus, protein, riboflavin, vitamin B12, and zinc was close to zero for the population group meeting dairy food recommendations (Table 4).

When separated by age (children 2–18 years and adults 19+ years), a smaller percentage of children and adults meeting dairy food recommendations was below the EAR for calcium (*p* < 0.0001), magnesium (*p* < 0.0001), phosphorus (*p* < 0.0001), protein (adults only) (*p* < 0.0001), riboflavin (*p* < 0.0001), vitamin A (*p* < 0.0001), vitamin B12 (*p* = 0.0084 for children; *p* < 0.0001 for adults), vitamin D (*p* < 0.0001), and zinc (*p* < 0.001) compared to adults and children who did not meet dairy recommendations. In addition, a higher percentage of children and adults meeting dairy food recommendations was above AI for potassium (*p* < 0.0001) and choline (*p* < 0.0001) compared to those not meeting recommendations. Inadequate intake of calcium, phosphorus, protein, riboflavin, and vitamin B12 was close to zero (% below EAR) for both children and adults meeting dairy food recommendations (Table 5). More children and adults consuming below recommendations for dairy food intake exceeded added sugar intakes (*p* < 0.0001 for children; *p* = 0.0075 for adults), while adults and children meeting dairy recommendations were more likely to exceed the recommended intakes for saturated fat (*p* < 0.0001) and sodium (*p* = 0.0343 for children; *p* < 0.0001 for adults).

A significantly smaller percentage of both females and males meeting dairy food recommendations was below the EAR for calcium (*p* < 0.0001), magnesium (*p* < 0.0001), phosphorus (*p* < 0.0001), protein (*p* < 0.0001), riboflavin (*p* < 0.0001), vitamin A (*p* < 0.0001), vitamin B12 (*p* < 0.0001), vitamin D (*p* < 0.0001), selenium (*p* = 0.0001 for females; *p* = 0.0246 for males), and zinc (*p* < 0.0001) compared to those who did not meet dairy recommendations (Table 6). Furthermore, a larger percentage of females and males meeting dairy food recommendations was above the AI for potassium and choline compared to those not meeting recommendations (*p* < 0.0001 for both). Population inadequacy (% below EAR) for calcium, phosphorus, protein, riboflavin, selenium, zinc, and vitamin B12 was also close to zero for both females and males meeting dairy food recommendations. Females and males meeting dairy food recommendations were more likely to exceed 10% of calories from saturated fat (*p* < 0.0001). Females meeting dairy recommendations were less likely to exceed 10% of energy from added sugars than females consuming below recommendations for dairy food intake (*p* = 0.0003).

Results were similar when nutrient intake and dairy food intake were assessed by ethnicity and race (Table 7). A smaller percentage of Hispanics, non-Hispanic Blacks, non-Hispanic Whites, and non-Hispanic Asians meeting dairy recommendations was below EAR for calcium (*p* < 0.0001), magnesium (*p* < 0.0001), phosphorus (0.0016 for non-Hispanic Asians; *p* < 0.0001 for all other groups), protein (*p* = 0.0008 for Hispanics; *p* = 0.0114 for non-Hispanic Asians; *p* < 0.0001 for both other groups), riboflavin (*p* = 0.0002 for non-Hispanic Asians; *p* < 0.0001 for all other groups), vitamin A (*p* < 0.0001), vitamin B12 (*p* = 0.0003 for non-Hispanic Blacks; *p* = 0.0001 for Hispanics; *p* < 0.0001 for both other groups), vitamin D (*p* < 0.0001), and zinc (*p* < 0.0001) compared to those not meeting dairy recommendations. A larger percentage of Hispanics, non-Hispanic Blacks, non-Hispanic Whites and non-Hispanic Asians meeting dairy food recommendations was also above AI for potassium and choline (*p* < 0.0001) compared to individuals from those same groups not meeting dairy food recommendations. Population inadequacy (% below EAR) for calcium, phosphorus, protein, riboflavin, vitamin B12, and zinc was close to zero for Hispanics, non-Hispanic Blacks, non-Hispanic Whites, and non-Hispanic Asians meeting dairy food recommendations. Hispanics and non-Hispanic Whites meeting dairy recommendations were more likely to exceed the AI for sodium (*p* < 0.0001), and Americans across racial and ethnic groups who met dairy recommendations exceeded 10% of calories from saturated fats (*p* < 0.0001). 

## 4. Discussion

The percentage of the American population meeting dairy recommendations was more likely to be above the EAR for a range of nutrients and the AI for potassium and choline. Most children and adults in this study who met recommendations for dairy foods had intakes above the EAR for calcium, magnesium, phosphorus, protein, riboflavin, vitamin A, and vitamin B12, and over 70% of Americans who met recommendations for dairy foods also met the AI for potassium. The nutrient adequacy results were similar across age, sex, and ethnic/racial groups. American adults meeting dairy food recommendations were also more likely to have adequate intake of protein than adults not meeting dairy recommendations, though this difference was not significant in children. Population inadequacy (% below EAR) was close to zero for calcium, phosphorus, riboflavin, and vitamin B12 among Americans meeting dairy food recommendations across all age, sex, and ethnic/racial groups. In contrast, nearly 60% of Americans ages 2 years and older who did not meet dairy food recommendations consumed calcium and magnesium below the EAR, and only 20% of Americans not meeting dairy recommendations were above the AI for potassium [17]. Results of this study indicate that it can be difficult to meet nutrient needs without adequate consumption of dairy foods.

In addition to being recommended by the DGA, dairy foods are part of healthy eating patterns recommended by the American College of Cardiology and the American Heart Association [18,19,20], the National Osteoporosis Foundation [21], and the American Academy of Pediatrics [22]. Results of this study indicate that the U.S. population still largely does not meet recommendations for dairy food intake. Some groups especially at risk for underconsuming dairy foods include females (children and adults), older children (9–18 years), older adults, non-Hispanic blacks, non-Hispanic Asian adults, children below a poverty-income ratio of 1.35, and Americans with overweight or obesity.

Actual or perceived lactose intolerance may be a reason that some Americans, including non-Hispanic blacks and non-Hispanic Asians, do not meet recommendations for dairy intake [23]. A consensus statement published following a 2010 National Institutes of Health Conference on Lactose Intolerance and Health indicates that individuals with lactose intolerance, whether actual or perceived, may be predisposed to “decreased bone accrual, osteoporosis, and other adverse health outcomes” as a result of insufficient dairy food and calcium intake [6]. A 2013 joint consensus statement on lactose intolerance from the National Medical Association and the National Hispanic Medical Association further indicated that some minority groups consume fewer dairy foods than the general population and are at a higher risk for developing chronic disease conditions such as hypertension and diabetes [24], which are associated with low calcium intake from dairy foods [25]. However, most individuals can continue to consume dairy foods while avoiding symptoms of lactose intolerance. A systematic review indicates that individuals with lactose intolerance can tolerate about 12 g of lactose—the amount in one cup of milk—especially when it is consumed with other foods [26,27]. Management strategies, such as selecting lactose-free milk and choosing yogurt with live and active cultures, can also help people with lactose intolerance to meet recommendations for dairy intake [6,27,28]. Lactose-free cow’s milk is an increasingly popular option for individuals with lactose intolerance, is recommended in the 2015 DGA as an option for meeting dairy recommendations, and can be found in 18% of U.S. households and 96% of U.S. retail food outlets [29,30,31].

Though dairy foods are a low-cost source of important shortfall nutrients for Americans [32], cost may also be a factor contributing to different nutrient intake among some groups. Children from households with a PIR < 1.35 were more likely to consume fewer servings of dairy foods than the DGA recommends. While the DGA takes many factors into account when developing healthy eating patterns, diet cost and food accessibility, including food cost, are not part of those considerations. While over 94% of retail outlets (food stores, drugstores, and convenience stores) in the U.S. sell one or more types of dairy food and most households with children purchase some dairy foods [33], cost and perishability of dairy foods may still prohibit some families from purchasing adequate amounts of dairy foods to enable all family members to meet recommendations. Market research data from early 2020 (through 23 February 2020) [34] indicate that a serving of low-fat milk may cost as little USD 0.18 from a private label gallon container, but the cost rises to USD 0.66 per serving, on average, for low-fat milk purchased by the half gallon. In addition, individuals needing lactose-free or reduced-lactose dairy products may also have challenges accessing adequate dairy foods, as these products also tend to be more expensive [34]. While dairy foods may be physically accessible and a low-cost source for important nutrients, they may not be sufficiently financially accessible for some Americans—an obstacle to adequate intake.

Americans with overweight or obesity are also at higher risk of underconsuming dairy foods, according to results of this study. While there are many potential reasons that this group may not be meeting recommendations for dairy intake, one reason may be due to a perception that consuming dairy foods is linked with increased adiposity. In this study, children and adults meeting dairy food recommendations tended to report higher energy intakes but lower BMI z-scores or BMIs. This finding aligns with several other studies in the literature, indicating that consuming dairy foods in recommended amounts is not linked with increased risk of adiposity in children or adults. For instance, two meta-analyses [35,36] and a systematic review [37] as well as several prospective cohort studies and cross sectional studies [38,39,40,41,42] indicate that dairy intake is inversely associated with or not associated with markers of adiposity in children and adolescents. In those reviews and studies, drinking plain milk was not associated with BMI or BMI z-scores in children, though it increased caloric intake and energy density at meals. A systematic review [36] and a randomized controlled trial [43] also indicate that drinking milk is not linked to increased risk of overweight and obesity in adults. Finally, the Scientific Report of the 2020–2025 Dietary Guidelines Advisory Committee (DGAC) notes that consuming low-fat dairy foods as part of a healthy eating pattern has beneficial impacts on outcomes related to growth, size, body composition, and obesity in adults (moderate evidence) and children (limited evidence) [3]. The Scientific Report of the 2020 DGAC also found limited evidence to suggest that milk intake was not associated with adiposity in either children or adults [3].

This study also indicates that Americans who consumed adequate amounts of dairy foods were more likely to consume more sodium and calories from saturated fat and, for some groups, fewer calories from added sugars. The average sodium intake of Americans ages 2+, according to a publication from the National Academies of Science, Engineering, and Medicine, was approximately 3400 mg/day, which exceeds the sodium AI (1500 mg/day for ages 14+) [44]. In this study, Americans ages 2+ who did not meet dairy food recommendations consumed less sodium, averaging approximately 3200 mg/day of sodium compared to the over 4400 mg/day consumed by Americans who did meet dairy food recommendations. The only groups in this study without significant differences in percent population meeting dairy recommendations and exceeding the AI for sodium were males (*p* = 0.0517), non-Hispanic blacks (*p* = 0.7372), and non-Hispanic Asians (*p* = 0.8152). In this study, individuals meeting dairy recommendations were more likely to consume above 10% of their calories from saturated fat regardless of age (children, adults), gender (females and males), and racial or ethnic background (Hispanics, non-Hispanic Blacks, non-Hispanic Whites, non-Hispanic Asians).

The relationship between meeting dairy recommendations and added sugar intakes was less consistent. Among all Americans over the age of 2, more individuals consuming less than recommended dairy intakes consumed more than 10% of their calories from added sugars (*p* = 0.0021). This association was not significant across all population segments. Hispanics, non-Hispanic Whites, and non-Hispanic Asians, females, children, and adults had significant relationships between added sugars intake and dairy intake. Children and non-Hispanic Asians meeting dairy recommendations were more likely to exceed 10% of calories from added sugars, but for all other population groups, those meeting dairy recommendations were less likely to exceed 10% of calories from added sugars. There was not a significant difference in the percent of the population exceeding added sugar recommendations between those meeting and those not meeting dairy recommendations among males and non-Hispanic Blacks.

The relationships between adequate dairy intake and elevated consumption of sodium and saturated fats and, for some population groups, added sugars, observed in this study, indicate a need for consumers to receive additional education around DGA recommendations. Consuming adequate servings of dairy foods while staying within recommended limits for sodium, added sugars, and saturated fat is possible, as demonstrated in food pattern modeling presented in both the 2015 DGA [1] and the Scientific Report of the 2020 Dietary Guidelines Advisory Committee [3]. Consumers may need additional guidance to follow the recommended healthy eating patterns and implement recommendations in the DGA.

Finally, while this study used a large nationally representative data sample, it also had some important limitations. Some limitations include the cross-sectional study design of NHANES, which prevents the determination of any cause and effect relationship. In addition, NHANES relies on self-reported dietary intake date, which may over- or underrepresent actual intake on an individual basis. It is also difficult to completely account for residual confounding (e.g., those who met recommendations might have also consumed more fruits and vegetables or other types of foods); thus, the improvements in nutrient intakes cannot be ascribed to dairy foods alone. Furthermore, the data used in this study were limited to foods available in NHANES 2013–2016, which may not reflect all food options currently in the marketplace, and we did not attempt to characterize the food source of the dairy intake, which may have been milk, cheese, yogurt, or other dairy foods, which could impact the results.

## 5. Conclusions

Although it may be possible for Americans to meet their nutrient needs without dairy foods, evidence from NHANES about how Americans actually eat indicates that dairy foods make important and unique contributions to dietary patterns, contributing calcium, magnesium, phosphorus, riboflavin, vitamin A, vitamin B12, zinc, potassium, and choline. Those who are not eating enough dairy foods are not consuming sufficient amounts of these nutrients from other sources. Those Americans who do meet current recommendations for dairy food intake have improved nutrient adequacy for several important shortfall nutrients.

## Figures and Tables

**Table 1:** Dairy intake recommendations from the 2015–2020 Dietary Guidelines for Americans.

Age Range	Recommended Dairy Intake (in Cup-Equivalents)
2–3 years	2
4–8 years	2.5
9 year and older	3

**Table 2:** Demographic characteristics of U.S. subpopulations consuming above recommended daily servings of dairy foods and those consuming fewer than the recommended daily servings of dairy foods in the National Health and Nutrition Examination Survey 2013–2016 ^1^.

Variables	Children 2–18 Years			Adults 19+ Years		
	Below RS ^2^ (*n* = 4212)	Above RS ^2^ (*n* = 1458)	*p*-Value ^3^	Below RS (*n* = 8934)	Above RS (*n* = 1178)	*p*-Value
Age (Years)	10.7 ± 0.1	8.9 ± 0.2	<0.0001	48.3 ± 0.4	44.0 ± 0.8	<0.0001
Gender: Male (%)	47.0 ± 1.0	60.5 ± 1.5	<0.0001	46.6 ± 0.6	67.5 ± 1.7	<0.0001
Race/Ethnicity						
Hispanic (%)	24.5 ± 3.1	22.9 ± 3.0	0.2862	15.1 ± 1.7	14.8 ± 2.1	0.7930
Non-Hispanic White (%)	50.2 ± 4.0	56.2 ± 4.0	0.0026	63.3 ± 2.5	74.8 ± 2.4	<0.0001
Non-Hispanic Black (%)	15.7± 2.2	10.2 ± 1.8	0.0001	12.1 ± 1.4	5.5 ± 1.1	<0.0001
Non-Hispanic Asian (%)	4.6 ± 0.8	4.6 ± 0.8	0.9331	6.0 ± 0.8	2.6 ± 0.5	<0.0001
Poverty Income Ratio (PIR)						
PIR < 1.35 (%)	35.8 ± 2.6	31.3 ± 3.2	0.0403	23.8 ± 1.4	24.1 ± 2.2	0.8039
1.35 <= PIR <= 1.85 (%)	11.2 ± 0.9	11.2 ± 1.7	0.9543	10.4 ± 0.6	10.2 ± 1.6	0.9070
PIR > 1.85 (%)	53.0 ± 2.9	57.5 ± 3.5	0.0913	65.9 ± 1.7	65.7 ± 3.2	0.9496
Physical Activity Level						
Sedentary	12.5 ± 0.9	10.6 ± 1.5	0.2326	22.4 ± 0.8	18.8 ± 1.9	0.0618
Moderate	23.8 ± 1.2	18.7 ± 1.3	0.0106	36.3 ± 0.7	31.7 ± 2.0	0.0268
Vigorous	63.7 ± 1.6	70.7 ± 1.8	0.0019	41.2 ± 0.9	49.4 ± 2.4	0.0013
Weight Status						
Overweight (%)	17.9 ± 0.6	13.2 ± 0.9	0.0002	32.5 ± 0.6	33.2 ± 2.2	0.7501
Overweight or Obese (%)	36.7 ± 1.4	28.3 ± 1.5	<0.0001	71.9 ± 0.8	67.0 ± 1.7	0.0034
Obese (%)	18.9 ± 1.2	15.0 ± 1.3	0.0082	39.5 ± 0.9	33.8 ± 2.3	0.0162

^1^ Data are means and standard errors using Day 1 dietary recall data. ^2^ Below RS: below recommended servings of dairy; ^3^ Above RS: at or above recommended servings of dairy. ^3^ P-value represents t-test comparing Below RS vs. Above RS. A cup-equivalent of dairy foods is 1 cup of milk, yogurt, or fortified soymilk; 1.5 ounces of natural cheese; or 2 ounces of processed cheese. Poverty Income Ratio (PIR).

**Table 3:** Energy intake, grams of food intake, and Body Mass Index of U.S. population 2+ years consuming above recommended daily servings of dairy and those consuming fewer than the recommended daily servings of dairy in the National Health and Nutrition Examination Survey 2013–2016 ^1^.

Additional Demographics	Population below Dairy Recommendations	Population above Dairy Recommendations
Average Energy Intake		
Kilocalories (kcal)	1926 ± 12	2733 ± 41
Amount of Food Consumed		
Food (g)	3093 ± 35	3553 ± 67
Body Mass Index (BMI) Z-score/BMI		
BMI z-score (ages 2–18 years)	0.59 ± 0.04	0.46 ± 0.04
BMI (ages 19+ years)	29.4 ± 0.2	28.3 ± 0.3

^1^ Data are means and standard errors using Day 1 dietary recall data. Body Mass Index (BMI).

**Table 4:** Percent U.S. population (ages 2+, male and female combined) below the Estimated Average Requirement or Above Adequate Intake among those consuming above recommended daily servings of dairy and those consuming fewer than the recommended daily servings of dairy in the National Health and Nutrition Examination Survey 2013–2016 ^1^.

Nutrients	% Population below Estimated Average Requirement		
	Below Dairy Recommendations (*n* = 13,146)	Above Dairy Recommendations (*n* = 2636)	*p*-Value ^2^
Calcium	63.3 ± 0.8	0.05 ± 0.07	<0.0001
Magnesium	57.1 ± 1.1	14.4 ± 1.2	<0.0001
Phosphorus	6.5 ± 0.6	0.01 ± 0.01	<0.0001
Protein	2.3 ± 0.4	0.0 ± 0.0	<0.0001
Riboflavin	3.5 ± 0.4	0.01 ± 0.004	<0.0001
Vitamin A	50.9 ± 1.6	3.4 ± 1.2	<0.0001
Vitamin B12	5.5 ± 0.7	0.01 ± 0.02	<0.0001
Vitamin D (D2 + D3)	98.5 ± 0.3	59.7 ± 2.3	<0.0001
Selenium	0.6 ± 0.2	0.0 ± 0.0	<0.0001
Zinc	22.0 ± 1.1	0.2 ± 0.1	<0.0001
	% Population above Adequate Intake/Recommended Intakes		
Sodium	98.9 ± 0.2	100 ± 0.0	<0.0001
Potassium	21.2 ± 0.9	71.5 ± 2.0	<0.0001
Choline	6.5 ± 0.6	36.9 ± 2.3	<0.0001
Energy from saturated fat	65.8 ± 1.4	98.1 ± 0.7	<0.0001
Energy from added sugars	64.0 ± 1.1	56.6 ± 2.2	0.0021

^1^ Data are means and standard errors using usual intake estimated with the National Cancer Institute method. ^2^ *p*-value assessed using z-score comparing percentages for below dairy recommendations to percentages for at/above dairy recommendations.

**Table 5:** Percent U.S. population (by age group) below the Estimated Average Requirement or Above Adequate Intake among those consuming above recommended daily servings of dairy (Above RS) and below recommended daily servings of dairy (Below RS), in the National Health and Nutrition Examination Survey 2013–2016 ^1^.

Nutrients	% Population below Estimated Average Requirement					
	Children 2–18 Years			Adults 19+ Years		
	Below RS ^2^ (*n* = 4212)	Above RS ^3^ (*n* = 1458)	*p*-Value ^4^	Below RS (*n* = 8934)	Above RS (*n* = 1178)	*p*-Value
Calcium	77.0 ± 1.2	0.1 ± 0.2	<0.0001	59.6 ± 1.0	0.01 ± 0.01	<0.0001
Magnesium	45.3 ± 1.5	10.8 ± 1.8	<0.0001	60.1 ± 1.2	16.8 ± 1.9	<0.0001
Phosphorus	29.2 ± 2.4	0.03 ± 0.04	<0.0001	0.8 ± 0.2	0.0 ± 0.0	<0.0001
Protein	1.5 ± 0.8	0.0 ± 0.0	0.0615	2.5 ± 0.4	0.0 ± 0.0	<0.0001
Riboflavin	1.9 ± 0.8	0.0 ± 0.0	0.0179	3.8 ± 0.4	0.01 ± 0.01	<0.0001
Vitamin A	38.5 ± 2.5	0.3 ± 0.2	<0.0001	54.1 ± 1.5	5.4 ± 1.9	<0.0001
Vitamin B12	2.6 ± 1.0	0.0 ± 0.0	0.0084	6.9 ± 0.8	0.01 ± 0.03	<0.0001
Vitamin D	99.5 ± 0.2	57.4 ± 2.8	<0.0001	98.3 ± 0.4	60.8 ± 2.9	<0.0001
Selenium	0.2 ± 0.2	0.0 ± 0.0	0.24	0.7 ± 0.2	0.0 ± 0.0	<0.0001
Zinc	17.7 ± 2.2	0.1 ± 0.1	<0.0001	23.0 ± 1.1	0.3 ± 0.2	<0.0001
	% Population above Adequate Intake/Recommended Intakes					
Sodium	99.7 ± 0.2	100 ± 0.0	0.0343	98.8 ± 0.2	100 ± 0.01	<0.0001
Potassium	14.0 ± 1.6	70.3 ± 2.7	<0.0001	23.1 ± 1.1	72.1 ± 2.5	<0.0001
Choline	10.6 ± 1.3	48.4 ± 2.6	<0.0001	5.5 ± 0.7	30.2 ± 2.7	<0.0001
Energy from saturated fat	72.2 ± 2.9	98.3 ± 1.1	<0.0001	64.0 ± 1.7	98.0 ± 1.0	<0.0001
Energy from added sugars	82.5 ± 2.7	64.6 ± 2.8	<0.0001	59.4 ± 1.0	52.0 ± 2.6	0.0075

^1^ Data are means and standard errors using usual intake estimated with the National Cancer Institute method. ^2^ Below RS: below recommended servings of dairy ^3^ Above RS: at or above recommended servings of dairy ^4^ *p*-value assessed using z-score comparing percentages for below dairy recommendations to percentages for at/above dairy recommendations.

**Table 6:** Percent U.S. population (by sex) below the Estimated Average Requirement or Above Adequate Intake among those consuming above recommended daily servings of dairy (Above RS) and below recommended daily servings of dairy (Below RS), ages 2+, in the National Health and Nutrition Examination Survey 2013–2016 ^1^.

Nutrients	% Population below Estimated Average Requirement					
	Females			Males		
	Below RS ^2^ *n* = 6969	Above RS ^3^ *n* = 1002	*p*-Value ^4^	Below RS *n* = 6177	Above RS *n* = 1634	*p*-Value
Calcium	73.5 ± 0.9	0.1 ± 0.1	<0.0001	51.5 ± 1.2	0.02 ± 0.02	<0.0001
Magnesium	54.8 ± 1.3	11.5 ± 2.0	<0.0001	60.0 ± 1.3	16.1 ± 1.4	<0.0001
Phosphorus	8.5 ± 0.7	0.03 ± 0.03	<0.0001	4.3 ± 0.6	0.01 ± 0.01	<0.0001
Protein	2.8 ± 0.5	0.0 ± 0.0	<0.0001	1.6 ± 0.3	0.0 ± 0.01	<0.0001
Riboflavin	3.3 ± 0.5	0.0 ± 0.01	<0.0001	3.6 ± 0.6	0.0 ± 0.01	<0.0001
Vitamin A	45.1 ± 1.9	1.7 ± 1.0	<0.0001	57.6 ± 1.7	4.3 ± 1.6	<0.0001
Vitamin B12	8.4 ± 1.2	0.02 ± 0.04	<0.0001	2.4 ± 0.5	0.0 ± 0.01	<0.0001
Vitamin D	99.3 ± 0.2	67.7 ± 3.5	<0.0001	97.6 ± 0.6	55.0 ± 2.7	<0.0001
Selenium	1.1 ± 0.3	0.0 ± 0.0	0.0001	0.1 ± 0.1	0.0 ± 0.0	0.0246
Zinc	21.6 ± 1.4	0.09 ± 0.09	<0.0001	22.4 ± 1.4	0.3 ± 0.2	<0.0001
	% Population above Adequate Intake					
Sodium	98.1 ± 0.3	100 ± 0.01	<0.0001	99.9 ± 0.03	100 ± 0.0	0.0517
Potassium	22.9 ± 1.2	70.4 ± 2.7	<0.0001	19.3 ± 1.0	71.7 ± 2.3	<0.0001
Choline	4.7 ± 0.5	33.5 ± 2.9	<0.0001	8.7 ± 1.0	39.2 ± 2.9	<0.0001
Energy from saturated fat	65.5 ± 1.9	99.3 ± 0.6	<0.0001	66.0 ± 1.8	97.5 ± 1.0	<0.0001
Energy from added sugars	64.9 ± 1.4	54.0 ± 2.7	0.0003	63.0 ± 1.2	58.3 ± 2.8	0.1185

^1^ Data are means and standard errors using usual intake estimated with the National Cancer Institute method. ^2^ Below RS: below recommended servings of dairy ^3^ Above RS: at or above recommended servings of dairy ^4^ *p*-value assessed using z-score comparing percentages for below dairy recommendations to percentages for at/above dairy recommendations.

**Table 7:** Percent U.S. population (by ethnicity/race) below the Estimated Average Requirement or Above Adequate Intake among those consuming above recommended daily servings of dairy (Above RS) and below recommended daily servings of dairy (Below RS), ages 2+, in the National Health and Nutrition Examination Survey 2013–2016 ^1^.

Nutrients	% Population below Estimated Average Requirement											
	Hispanics			Non-Hispanic Blacks			Non-Hispanic Whites			Non-Hispanic Asians		
	Below RS ^2^ *n* = 3787	Above RS ^3^ *n* = 811	*p*-Value ^4^	Below RS *n* = 3035	Above RS *n* = 404	*p*-Value	Below RS *n* = 4430	Above RS *n* = 1089	*p*-Value	Below RS *n* = 1325	Above RS *n* = 183	*p*-Value
Calcium	58.6 ± 1.3	0.0 ± 0.03	<0.0001	74.3 ± 1.7	0.0 ± 0.7	<0.0001	61.6 ± 1.1	0.05 ± 0.07	<0.0001	76.2 ± 3.8	0.01 ± 0.2	<0.0001
Magnesium	52.7 ± 1.8	12.4 ± 2.0	<0.0001	66.6 ± 1.3	14.3 ± 2.3	<0.0001	57.9 ± 1.5	15.0 ± 1.6	<0.0001	49.4 ± 3.0	10.6 ± 3.7	<0.0001
Phosphorus	7.7 ± 0.9	0.01 ± 0.01	<0.0001	9.9 ± 0.8	0.0 ± 0.01	<0.0001	5.5 ± 0.7	0.01 ± 0.03	<0.0001	4.0 ± 1.3	0.01 ± 0.08	0.0016
Protein	1.6 ± 0.5	0.0 ± 0.01	0.0008	3.6 ± 0.7	0.0 ± 0.0	<0.0001	2.4 ± 0.6	0.0 ± 0.0	<0.0001	1.3 ± 0.4	0.05 ± 0.2	0.0114
Riboflavin	4.5 ± 0.9	0.0 ± 0.0	<0.0001	7.0 ± 1.2	0.0 ± 0.01	<0.0001	2.0 ± 0.4	0.02 ± 0.02	<0.0001	5.1 ± 1.4	0.0 ± 0.02	0.0002
Vitamin A	58.5 ± 2.3	1.0 ± 1.0	<0.0001	59.3 ± 2.1	2.8 ± 2.3	<0.0001	47.0 ± 2.3	4.3 ± 1.7	<0.0001	48.8 ± 2.9	0.5 ± 2.2	<0.0001
Vitamin B12	6.4 ± 1.6	0.02 ± 0.05	0.0001	4.7 ± 1.3	0.0 ± 0.01	0.0003	5.2 ± 1.0	0.01 ± 0.02	<0.0001	11.5 ± 2.3	0.0 ± 0.0	<0.0001
Vitamin D	98.9 ± 0.3	65.1 ± 6.5	<0.0001	99.6 ± 0.2	69.1 ± 5.1	<0.0001	98.4 ± 0.4	58.2 ± 3.1	<0.0001	95.5 ± 1.3	40.8 ± 12.1	<0.0001
Selenium	0.5 ± 0.2	0.0 ± 0.0	0.0039	1.0 ± 0.4	0.0 ± 0.0	0.0143	0.8 ± 0.2	0.0 ± 0.0	0.0003	0.1 ± 0.1	0.03 ± 0.06	0.5076
Zinc	18.7 ± 2.0	0.08 ± 0.1	<0.0001	27.6 ± 1.6	0.4 ± 0.2	<0.0001	22.1 ± 1.7	0.3 ± 0.2	<0.0001	20.0 ± 3.1	0.6 ± 1.3	<0.0001
	% Population above Adequate Intake											
Sodium	99.0 ± 0.2	100 ± 0.0	<0.0001	99.5 ± 1.5	100 ± 0.02	0.7372	98.9 ± 0.3	100 ± 0.01	<0.0001	99.8 ± 0.2	99.6 ± 0.7	0.8152
Potassium	22.7 ± 1.6	72.4 ± 3.2	<0.0001	10.6 ± 3.5	90.1 ± 16.0	<0.0001	19.7 ± 3.2	71.0 ± 2.7	<0.0001	24.8 ± 2.7	83.2 ± 8.5	<0.0001
Choline	8.6 ± 0.9	46.8 ± 3.7	<0.0001	7.8 ± 1.1	51.7 ± 10.0	<0.0001	5.8 ± 0.9	32.7 ± 3.1	<0.0001	7.6 ± 1.5	52.7 ± 8.1	<0.0001
Energy from saturated fat	58.6 ± 2.5	98.4 ± 1.7	<0.0001	60.9 ± 3.1	99.1 ± 1.3	<0.0001	72.3 ± 2.3	98.3 ± 0.9	<0.0001	31.1 ± 2.2	80.5 ± 6.7	<0.0001
Energy from added sugars	68.1 ± 1.8	49.5 ± 3.7	<0.0001	76.1 ± 1.9	73.3 ± 4.6	0.5759	63.0 ± 1.6	56.8 ± 2.5	0.0346	28.8 ± 2.7	43.2 ± 5.0	0.0102

^1^ Data are means and standard errors using usual intake estimated with the National Cancer Institute method. ^2^ Below RS: below recommended servings of dairy; ^3^ Above RS: at or above recommended servings of dairy ^4^ *p*-value assessed using z-score comparing percentages for below dairy recommendations to percentages for at/above dairy recommendations.
